# Nitric Oxide Analysis Down to ppt Levels by Optical-Feedback Cavity-Enhanced Absorption Spectroscopy

**DOI:** 10.3390/s18071997

**Published:** 2018-06-22

**Authors:** Lucile Richard, Daniele Romanini, Irène Ventrillard

**Affiliations:** LIPhy, University Grenoble Alpes, CNRS, F-38000 Grenoble, France; lucile.richard@univ-grenoble-alpes.fr (L.R.); daniel.romanini@univ-grenoble-alpes.fr (D.R.)

**Keywords:** nitric oxide, trace gas sensor, infrared laser spectroscopy, cavity enhanced spectroscopy, interband cascade laser, real-time monitoring

## Abstract

Monitoring nitric oxide at the trace level is required in a large range of applications. We report on a trace gas analyzer optimized for nitric oxide measurements by Optical Feedback Cavity Enhanced Absorption Spectroscopy with an interband cascade laser at 5.3 µm. The short response time of the instrument allows for reaching the level of 50 ppt in only 180 ms. Its stability enables averaging up to 12 min to reach a detection limit of 0.9 ppt. Absolute concentration calibration requires to account for the optical saturation effect that results from the intense absorption line intensity addressed here, in the mid infrared region, in contrast to instruments that are operating in the near infrared region.

## 1. Introduction

Nitric oxide (NO) is a molecule of high interest at the trace level in a large range of applications. It is the most abundant of the nitrogen oxides (NO_x_) that are involved in the atmospheric ozone cycle [1]. Hence, despite its low concentration level in the atmosphere (ranging from a few ppb down to several ppt in very remote and unpolluted areas), NO plays a major role in environmental monitoring, atmospheric chemistry, and in the study of air-snowpack interaction processes [2]. In medicine, NO is a signaling molecule in different physiological mechanisms of the cardiovascular system, the nervous system, and the immune system [3]. In exhaled breath, NO constitutes a major marker of airway inflammation diseases and lung diseases [4]. As an example, it is routinely monitored in asthmatic patients. Monitored NO concentration levels in exhaled breath of healthy patients range typically from 1 to 10 ppb.

As a consequence of the large range of applications, several laser spectrometers that are dedicated to NO trace detection have been developed [5,6,7,8,9,10,11,12,13,14,15]. To obtain a sub-ppb sensitivity by direct measurement, strong absorption lines in the mid-infrared region (MIR) need to be addressed. But, in this spectral region, lasers, detectors, and optical components are not as well developed as in the near infrared region (NIR). In particular, all of the previously developed NO laser spectrometers reaching sub-ppb sensitivity [8,9,10,11,12,13] exploit continuous wave quantum cascade lasers (CW-QCL) needing a thermal dissipation around 5–10 W, which poses a challenge to the development of compact field instruments. Nonetheless, some of these instruments are transportable. The best performance of in-situ measurements is reported for lower troposphere measurements in a high altitude alpine station with an analyzer that is optimized from a commercialized instrument by Aerodyne Research Inc. (Billerica, MA, USA). A detection limit of 7 ppt is reached with an averaging time of 80 s (45 ppt in 1 s) [12,16]. This instrument operates with a multi-pass optical cell combined with laser wavelength modulation. It requires careful and regular calibration together with a thermoelectric liquid chiller used for temperature stabilization of the entire optical set-up additionally to the required laser water cooling. While this instrument is designed for in-situ measurements, it is quite bulky, with a total weight of 175 kg. Operating with a different technique (off-axis integrated cavity output spectroscopy), a more compact instrument (still weighting 68 kg) is commercially available from Los Gatos Research Inc. (San Jose, CA, USA). Its slightly lower detection limit of 100 ppt in 10 s [11] remains well adapted to many in-situ measurements.

Some other instruments avoid the laser water cooling by indirect NO measurements. These are based on the conversion of NO to NO_2_ in excess ozone [17]. Then, NO_2_ trace detection can be performed in the near-UV spectral region where room temperature lasers are available. Such a spectrometer was developed for deployment on an aircraft. By using cavity ring-down spectroscopy (CRDS) with a continuous wave diode laser working at 405 nm, a detection limit of 20 ppt is reached within an averaging time of 60 s (140 ppt in 1 s) [14]. Indirect NO detection is also used in the well-established chemiluminescence instruments: NO reacts with O_3_ to form excited NO_2_ whose luminescence is monitored. Today, the best commercial chemiluminescence detectors that are available offer a detection limit of 50 ppt in 120 s (42i-TL analyzer from ThermoFischer Scientific, Waltham, MA, USA). Its response time of 120 s is compatible with most atmospheric monitoring measurements, but it is not sufficient to allow for a decent spatial resolution during airborne campaigns, nor to resolve the respiratory cycles for real-time breath analysis. A research-grade chemiluminescence instrument was developed more than 15 years ago [18], and it is still at the state of the art for NO detection, featuring a sensitivity of 5 ppt within 1 min averaging time (with 1 Hz acquisition frequency). This instrument has been used in remote areas, such as in Antarctica, where NO concentration can drop to a few ppt [2]. To be operated at their best level of performance, chemiluminescence instruments need careful periodic calibrations with referenced gas sample.

In this paper, we present a NO analyzer with direct measurements by Optical-Feedback Cavity Enhanced Absorption Spectroscopy (OF-CEAS) [19,20]. OF-CEAS allows for the development of robust and compact trace analyzers that are adapted to harsh field conditions [21,22] and airborne measurements [23,24,25]. In this work, for the first time, a NO analyzer is built using an Interband Cascade Laser (ICL). This kind of laser became recently available in the MIR and it has the major advantage of laser current-voltage characteristics that are rather similar to those of DFB diode lasers working in the NIR. As a result, contrary to QCLs, no water cooling is required. Additionally, this instrument benefits of laser control electronics developed in the past years for NIR OF-CEAS instruments.

One major advantage is that OF-CEAS allows for a high signal to noise ratio at the high finesse optical cavity output, partially compensating for the lower efficiency in the MIR as compared to the NIR of detectors and optics (in particular, the high reflectivity mirrors composing the cavity). As a result, the newly developed MIR NO analyzer has a sensitivity of 6 × 10^−10^/cm (in absorption units) for a single absorption spectrum. This is nearly equivalent to the best OF-CEAS instruments optimized over more than a decade in the NIR (smallest detectable absorption coefficient of ~5 × 10^−10^/cm [26]). Here, a single measurement performed in 180 ms, allows a detection limit of NO of 50 ppt. Furthermore, the stability of the instrument allows for averaging down to 0.9 ppt in 12 min. This limit of detection is more than a factor 5 below that of the best instruments that are developed for NO detection [16,18]. An additional advantage for remote measurements is that OF-CEAS measurements do not require periodic calibration with certified mixtures, since it uses CRDS normalization of the absorption scale to derive absolute absorption values [27]. Nonetheless, it will be shown that addressing large intensity absorption lines in the MIR, optical saturation of the molecular transitions has to be accounted for, which could possibly add long term measurement drift. We believe that this work may open the path for the industrialization of OF-CEAS NO trace analyzers with a detection limit in the ppt range.

## 2. OF-CEAS Set-Up

**Figure 1:**
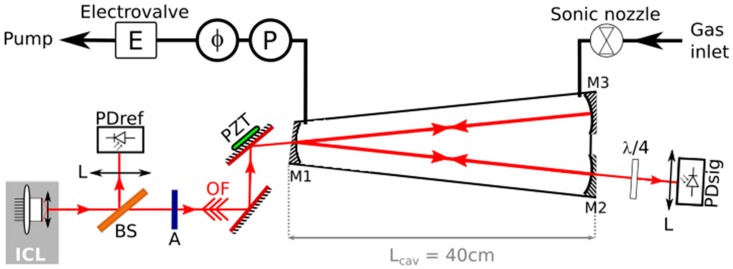
Experimental scheme of the Optical-Feedback Cavity Enhanced Absorption Spectroscopy (OF-CEAS) analyzer, using an ICL at 5.3 µm for nitric oxide (NO) detection. The V-cavity is made of three mirrors (M1, M2, and M3). A ZnSe beam splitter (BS) is used to monitor the laser power with the photodiode PDref. A glass microscope slide is used as an attenuator (A) to adjust the OF rate. OF phase is controlled by acting on a piezoelectric element (PZT) supporting one of the steering mirrors. A quarter-wave plate (λ/4) is used to minimize back reflection effect from the photodiode monitoring cavity transmission (PDsig). Pressure (P) and flux (φ) of the flowing gas are controlled.

As other Cavity Enhanced Absorption Spectroscopy (CEAS) techniques, OF-CEAS [19,20] relies on the use of a high finesse optical cavity as a gas cell, in order to increase the effective absorption path length. The peculiarity of this method is that the cavity is made of three mirrors in a V-shaped geometry (Figure 1) to avoid direct retro-reflection to the laser, while a fraction of the resonant optical field may return back to the laser. This optical feedback (OF) modifies the laser dynamics: the frequency of the laser emission locks to the frequency of the cavity resonances, (modes) and additionally, the laser spectral emission narrows down to a line width that is even smaller than the cavity mode width [28]. These modifications allow to obtain and record broad and intense cavity resonances as the laser frequency is scanned (see figure in the next section). As a result, OF-CEAS absorption spectra are retrieved with a typical signal to noise ratio exceeding 10^3^.

The experimental scheme of the newly developed OF-CEAS analyzer for NO trace detection is given in Figure 1. It is a typical set-up of an OF-CEAS analyzer, as described in several publications [13,19,27,29,30]. This instrument integrates an ICL (Nanoplus GmbH) that operates with single mode continuous wave emission and is tunable around 5.256 μm. It achieves this wavelength at an operating temperature of 13.8 °C, with an output power of 2 mW for an injection current of 55 mA. The laser is coupled to the V-cavity made of three wedged mirrors (LohnStar Optics) with a reflectivity of 99.986%, deduced from a cavity ring down value of 9.70 ± 0.03 µs for a cavity filled at a pressure of 100 mbar of dry nitrogen. The total length of the cavity is 80 cm (unfolded). Consequently, the cavity finesse is 11,400, while the Free Spectral Range (FSR) is equal to 187.5 MHz. To obtain the optimal OF rate [30], a glass microscope slide is used as an attenuator with about 20% transmission at 5.3 µm. A thermoelectrically cooled photodiode (VIGO SA) detects the cavity transmission (PDsig), while a room-temperature photodiode monitors the laser power (PDref). To minimize the optical fringes effects [19], lenses (L) are used to focalize light on the photodiodes in order to limit light scattering from the detectors edges. However, optical fringes are still observed in the base line of the absorption spectra, arising mainly from the scattered light from PDsig that couples back to the cavity. To minimize this effect, a quarter-wave plate (λ/4) is placed between the output of the cavity and PDsig. In this way, the polarization of the back scattered light after one round trip is mostly orthogonal to the cavity mode polarization and cannot couple to it (V-cavity modes of vertical and horizontal polarization are not degenerate in frequency). The elimination of this fringe can also be obtained by the fine adjustment of the cavity to photodiode distance to a multiple of the cavity length [31], however the use of a λ/4 plate is simpler and allows for a more compact set-up.

The gas to be analyzed is continuously flowing in the cavity. A flow-meter monitors the flux, which is fixed to 150 sccm by a sonic nozzle placed at the inlet of the gas line. A combination of an electro-valve and a pressure gauge allows for regulating the pressure to 107 mbar. In this configuration, the analyzer response time is limited by the internal volume of the cavity (~22 cm^3^) to about 1 s.

The control electronics fit in a single electronic card provided by AP2E, a company that exploits the OF-CEAS patent. In particular, it performs the laser thermal regulation and the generation of the laser current ramp. This card was initially developed for DFB diode lasers in the NIR and could be employed with minor changes for the ICL. On this card, an ARM9 processor is also used for the acquisitions of analogue signals from the photodiodes with a 16 bits digital converter and other sensors (pressure, flux, temperature), as well as the pressure regulation and the OF phase control (by controlling the PZT voltage). However, data analysis is computed in real-time by a home-made Labview routine.

At present, this instrument fits in a metal chassis that measures 90 × 60 × 35 cm^3^, for a total weight of about 35 kg (vacuum pump excluded). It is a laboratory set-up where optics are fixed with standard posts on a commercial breadboard. The next generation of this instrument will adopt the design of standard AP2E OF-CEAS analyzers in the NIR, which fit inside a standard 19 inch rack (45 × 59 × 13 cm^3^) with a total weight of about 15 kg.

## 3. Results

### 3.1. Limit of Detection (LOD)

**Figure 2:**
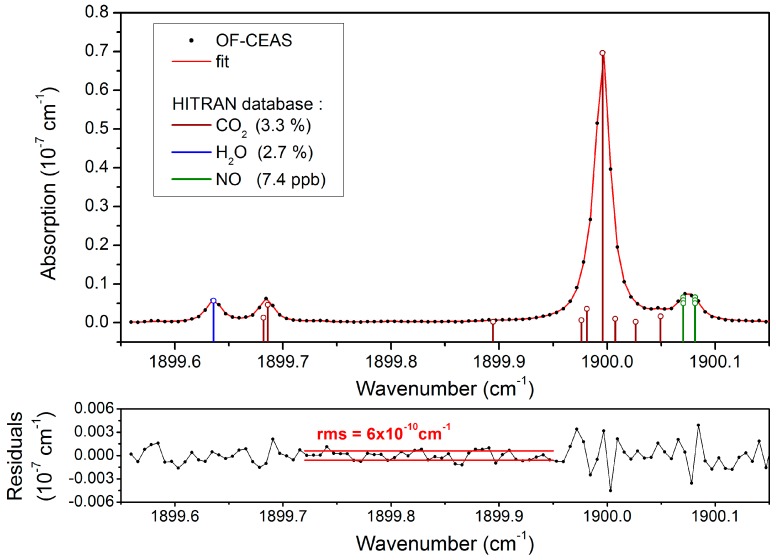
Top: OF-CEAS spectrum of exhaled air (black dots) and its fit (red line). It represents 110 spectral data points, recorded in 180 ms. Baseline and fringes are subtracted. Vertical sticks represent the absorption line derived from a HITRAN-based simulation. Note that each NO doublet line is actually the sum of three coinciding transitions. Given NO concentration is corrected for the saturation effect (see below). Bottom: Residuals of the spectral fit. On the complete scan, the rms is 1.3 × 10^−9^ cm^−1^, while on a region free of absorption line (in red) the rms is as low as 6 × 10^−10^ cm^−1^.

As most laser-based instruments for NO detection [8,9,10,11,12], the spectral region is chosen to exploit NO doublet absorption lines at 1900.08 cm^−1^ and 1900.07 cm^−1^ (Figure 2). These transitions are isolated from H_2_O and CO_2_ lines, which are the main interfering molecules for atmospheric analysis and breath analysis applications. The most demanding application concerning interferences is clearly breath analysis, where H_2_O and CO_2_ concentrations rise up to several percent. But, as observed in Figure 2 that shows a spectrum of exhaled breath, working at 107 mbar allows for well resolving the relatively strong CO_2_ line next to the NO doublet.

A single spectrum (Figure 2) is recorded in 180 ms. This time is currently limited by our electronics but it can be reduced by typically a factor 2. Indeed, the spectral scan width can be narrowed around NO (and CO_2_) lines, and the speed of the spectral scan can be increased as allowed by ICL dynamics [30]. Data analysis allows for directly obtaining the spectra in absolute absorption units by a ring-down measurement performed on the last cavity mode of each scan [27]. A non-linear fit [20] of the absorption line profiles is performed in order to obtain the concentration of each species present in the scan from the corresponding integrated line intensities. All of the absorption lines are fitted with a Voigt profile. According to the HITRAN [32] database, the NO doublet lines are specified to have both the same intensity and linewidth. Additionally, the baseline is included in the fit as a second order polynomial (in the basis of orthogonal Legendre Gauss polynomials). This baseline is not only fixed by the cavity losses (mirror transmission and losses), but is also due to absorption by far wings of molecular lines present around the fitted spectral window. In this spectral range, this continuum absorption is largely dominated by water absorption. The fit also allows for accounting for etalon fringes by adding sine waves at fixed frequency with free phase and amplitude [33]. Here one fringe is still detectable and is included in the fit, it is attributed to a residual of the back-scattering from PDsig (strongly damped by the λ/4). The concentration values are derived from the areas of the absorption lines computed by the fit procedure with a conversion factor obtained either from molecular absorption simulation with data base, such as HITRAN, or either from measurements of the calibrated gas sample [24]. The conversion factor has to be calculated for the pressure and temperature conditions of the experiment. We will show in the following, that due to optical saturation effect occurring in the MIR, the normalization procedure for NO concentration has to be adapted.

Figure 2 shows a typical spectrum of exhaled air by a healthy patient. The standard deviation of the fit residuals is 1.3 × 10^−9^ cm^−1^. Indeed, at high concentrations, such as here, this quantity is dominated by excess structured noise around the absorption lines. On the other hand, a correct estimation of the noise-equivalent absorption is obtained from a region without lines. It is two times lower (6 × 10^−10^ cm^−1^), as shown in the bottom of the Figure 2. This constitutes an improvement by a factor 3 as compared to our previous OF-CEAS setup that is dedicated to NO that was based on a QCL [13] (rms of 2 × 10^−9^ cm^−1^). The sensitivity, for a same noise on photodiodes, is inversely proportional to the ring-down time. In the ICL analyzer, despite a smaller optical cavity length than in the QCL instrument (0.8 m instead of 1 m), higher mirror reflectivity allows higher ring-down time (9.7 µs instead of 6.6 µs). Additional gain in the sensitivity can be attributed to the reduction of the fringes thanks to wedged mirrors and the quarter-wave plate (Figure 1).

**Figure 3:**
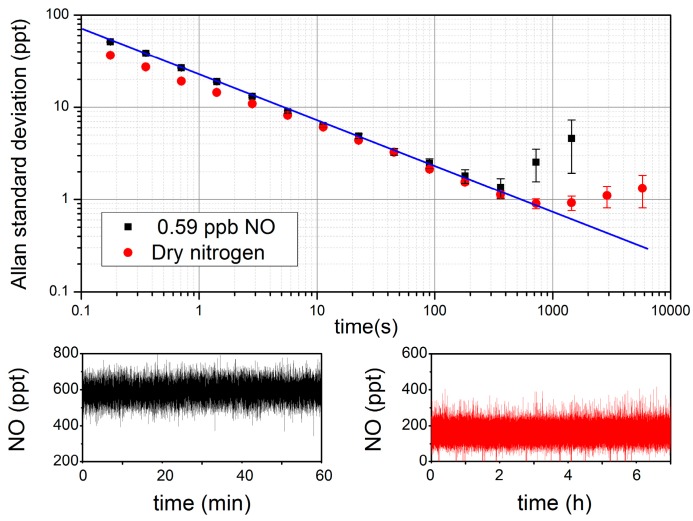
Top: Allan standard deviation derived from measurements shown at the bottom. In red, dry nitrogen (with residual traces of NO). In black, sample of 0.59 ppb of NO. The blue line represents the $1/\sqrt{t}$ slope expected from white noise averaging.

In order to analyze the stability and the detection limit of the instrument as a function of the averaging time, an Allan standard deviation [34] is performed on two samples (Figure 3). The first one (in black) is a sample of 0.59 ppb of NO that was obtained by dilution of a certified tank (191 ppb) in dry nitrogen (available from the laboratory gas line), while the second sample (in red) is dry nitrogen. These two measurements present similar results and indicate a Limit of Detection (LOD) of about 50 ppt for a single laser scan (180 ms). This level of sensitivity in such a short time was never reached before (and the acquisition rate could be even lowered in the future as explained before). Furthermore, the instrument is stable enough to average concentration values with white noise statistics during more than 12 min, achieving a LOD of 0.9 ppt. It is more than a factor 5 below that of the best instruments that were previously developed for NO trace detection [16,18].

### 3.2. Correction of Optical Saturation Effects

Optical saturation [35] can easily occur due to the strong optical field trapped in a high finesse cavity. Even more in the MIR region, where molecular transitions are more intense than in the NIR. When probing a strong transition with an intense laser field, the excited state may thus become significantly populated as a result of massive photon absorption winning the competition against other processes that remove molecules from the excited state (molecular collisions, walk-off from the light beam, spontaneous emission). Light absorption results from the difference between absorption from the lower to the excited state, and stimulated emission the other way round when the excited state is populated. The presence of a large light field leads, over relatively short time scales, to a steady state where absorption of molecules towards the excited state reaches an equilibrium with stimulated emission and other de-excitation processes. The main observable effect is induced transparency, i.e., a reduced light absorption by the sample. This optical saturation effect leads to a smaller apparent concentration being measured, and thus also to reduced sensitivity.

**Figure 4:**
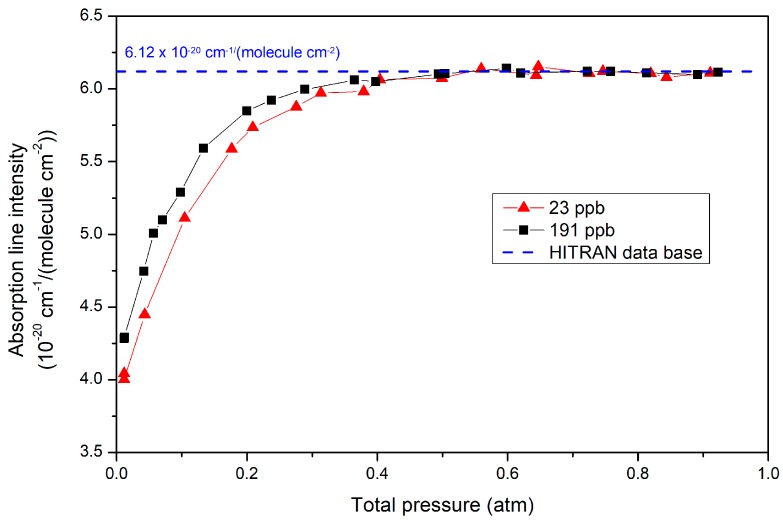
Evolution of NO absorption line intensity with pressure, for two different sample concentrations. One sample comes directly from a high-pressure tank (black squares), the other is obtained by flow dilution of gas from this tank with dry N_2_ (red triangles). As explained in the text, the concentration values are derived from a scaling parameter used to adjust high-pressure plateau values to the HITRAN value.

One way to illustrate this effect consists of computing the NO absorption line intensity (*S*) derived from measurements at different pressures (Figure 4). *S* is defined by the area under a single NO line (*A*) (derived from our fitting procedure) normalized by the density of absorbing NO molecules:

(1) $$S=\frac{A}{x_{i}P_{T}n_{0}\frac{T_{0}}{T}}$$

where *x_i_* is the mole fraction (named here “concentration”, as largely used by the community); *P_T_* the total pressure and *T* the temperature of the gas cell; and, *n_0_* is the Loschmidt constant defined at *T_0_* = 273.15 K. The dashed line in Figure 4, represents the expected value that is actually the sum of three NO transitions composing one of the doublet lines at 1900.07 cm^−1^. While *S* is expected to be constant, it is observed in Figure 4 that its values drop down for pressures below 0.4 atm. This illustrates the increase of optical saturation as the total pressure is reduced, resulting from a decrease of the collisional rate. On the other hand, constant *S* values that are obtained at high pressure indicate that collisions become completely dominant over optical pumping, with a plateau value that corresponds to the unsaturated line intensity reported in the HITRAN database (after a small adjustment of the sample concentration, as explained below).

In Figure 4, black squares represent measurements that are directly performed on a high-pressure tank mixture certified to 202 ppb ± 20 ppb of NO (Air-Liquide). While the accuracy specified for the line intensity in HITRAN is as well of 10%, we choose to adjust the unsaturated measurements (at high pressure) to the HITRAN value. This leads to a re-calibration of the tank value of 191 ± 19 ppb. Here and below, this concentration value is used in place of that given by the sample supplier (both values are anyway compatible). Indeed, we rather trust the result of high-quality fitting of the recorded absorption lines, with an instrumental error budget that is close to the 1% level (given the accuracy of pressure and temperature gauges). We actually find an almost perfect match of recorded spectra with HITRAN simulations performed at the same sample pressure and temperature in the high-pressure limit, with just a slight mismatch (~1%) of the linewidth, which could be attributed either to HITRAN parameters uncertainty or to a calibration error of our pressure gauge. This re-calibration also has the advantage of allowing for self-consistent calibration of future samples independently of an arbitrary commercial sample.

**Figure 5:**
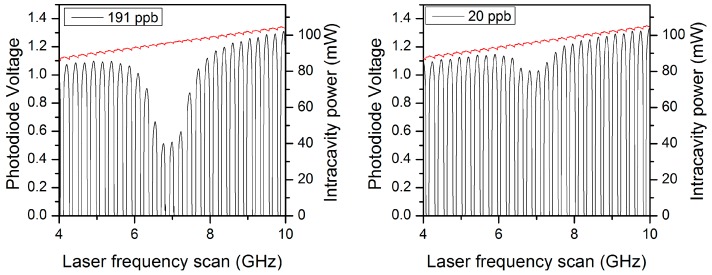
Zoom in the photodiodes signals recorded during the laser spectral scan around the NO absorption lines for two concentrations (at 107 mbar). The photodiode signals (in black) allows for monitoring the cavity transmission and to derive the intra-cavity power (right axis). Note that a small incident laser power change induced by the optical feedback (OF) is observed in the reference signal (in red).

Figure 4 also illustrates that in an OF-CEAS analyzer, the amount of saturation depends on the concentration. This is usually not accounted by models [36], but due to the absorption, the higher is the concentration the lower is the light intensity at the absorption line center. For example, as shown in Figure 5 around 100 mbar, 56% of the light intensity is absorbed at 191 ppb of NO, while it is only 13% at 20 ppb.

**Figure 6:**
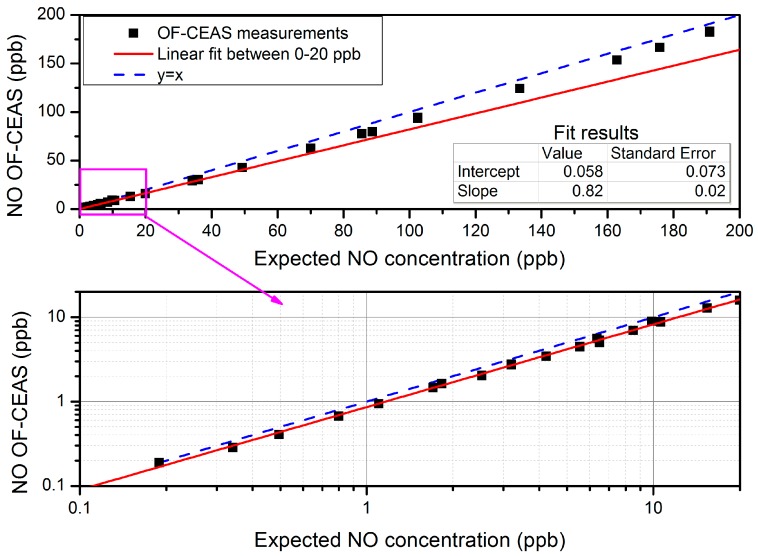
Preliminary to the correction of the optical saturation effect: Concentration given by the analyzer (NO OF-CEAS) versus the expected concentration from the dilution factor of a calibrated tank. The red line is a linear fit of the measurements between 0 and 20 ppb (shown in log-log scales in the bottom zoom for more clarity). Pressure in the cavity is regulated to 107 mbar.

In order to correct OF-CEAS concentration measurements from the effect of optical saturation, samples of different concentrations are measured when the analyzer is operated at its working pressure condition, close to 100 mbar. In Figure 6, measurements that were obtained from the surface of the fitted NO doublet line are plotted against expected values that were derived from the calibrated high-pressure value (191 ppb) and the dilution factor in dry nitrogen (using two mass flow controllers by Bronkhorst). It should be underlined that the line profiles are fitted with a Lorentzian broadening coefficient that is larger than expected at the given pressure (by a factor 1.15), which is a first evidence of saturation. In addition, the effect of optical saturation is apparent as a deviation from the expected y=x dependence (blue dashed line). Here again, it is observed that the saturation effect depends on NO concentration, being larger at lower concentration. Nevertheless, in the concentration range of 0 to 20 ppb (that covers both atmospheric monitoring and breath analysis applications), the saturation effect can be simply accounted for using a fixed scaling factor of 1.22 ± 0.02 derived from the linear fit in the range of interest (inverse of the slope of the red curve in Figure 6). This linear correction is valid for measurements up to 20 ppb as long as the given operation conditions are maintained (sample pressure, laser current ramp, and temperature). All of the concentrations given in this paper are corrected by this factor (expected in Figure 6). In particular, our setup allows for stable laser power levels and reproducible cavity injection efficiency as cavity output signals have negligible drift over a few weeks. It is also clear that a larger dynamic range (up to 200 ppb or more) could be readily covered by using a nonlinear correction curve fitted to all the data in Figure 6.

The absolute accuracy of this calibration is principally limited by the HITRAN accuracy of the absorption line intensity (10%) that is used to calibrate the high-pressure gas tank (to 191 ppb). Additional experimental uncertainty arises mainly from temperature and pressure gauges for the absolute measurement at the standard condition of operation, and flow meters calibration involved in the dilution of gas samples. All of these errors being independent and much smaller (below 1%), we should keep 10% as a global uncertainty estimate on the NO concentration measurements.

While the saturation effect can be corrected with a linear calibration for small concentrations, its theoretical modelling is not simple, even if we focus on the saturation at the line center, as is generally done [35]. Simple models allow for deriving the decrease of the absorption that scales as a function of the saturation parameter (*s*). When the Doppler broadening is dominant on the Lorenzian broadening (inhomogeneous broadening, observed in the regime of very low pressure) peak absorption scales as $1/\sqrt{\left(1+s\right)}$. On the other hand, it scales as 1/(1+s) in the homogeneous regime that is dominated by Lorentzian broadening (at higher pressure). The pressure regime of the OF-CEAS analyzer is fixed around 100 mbar. Basically, this regime represents a trade-off between sensitivity (related to the absorption value at the line center) and selectivity (related to the overlap of different absorption lines that depends on their linewidth). As a result, at the operating pressure, the inhomogeneous Doppler and homogeneous Lorentzian broadenings are comparable and none of the simple saturated absorption models are valid. Nonetheless, we estimate a saturation parameter of typically 0.22 for an experimentally estimated intra-cavity power of 80 mW (as observed at the line center for a concentration of 20 ppb, Figure 5). The inhomogeneous model would then predict an absorption decrease of 18% that is closer to the experimental measurements as compared to the inhomogeneous model (10% expected).

## 4. Conclusions

Recent ICLs developments open the path toward the development of a new generation of trace analyzers, in particular, given the fact that ICLs give access to strong molecular transitions in the MIR without the large power consumption and dissipation of QCLs. Here, an ICL tunable around 5.3 µm, is coupled with OF-CEAS technique to deliver a NO detection limit never achieved before, lower than 1 ppt, allowed by averaging during 10 min. This first demonstration with a laboratory set-up will be followed by the implementation in a more compact set-up that was previously designed for DFB diode laser in the NIR by the AP2E company (Aix-en-Provence, France) (instruments packaged in a standard 19 inch 4U rack). The reported sensitivity is adapted to the most stringent application, that is, the monitoring of NO in remote area where concentration drops below 10 ppt [2,37,38]. Furthermore, the short response time of the OF-CEAS analyzer offers a 50 ppt NO detection limit in only 180 ms. This can be exploited in different applications, such as aero-ported campaigns with enhanced spatial resolution or on-line breath analysis resolving the details of respiratory phases.

This detection limit is obtained by addressing intense absorption transitions in the MIR (*S*~6 × 10^−20^ cm^−1^/(molecule·cm^−2^)) combined with the use of a high finesse (F~10^4^) optical cavity to enhance the absorption path-length. In this configuration, optical saturation is observed given that relatively high laser-power levels are necessary inside the optical cavity (~100 mW) to provide enough optical feedback for laser frequency locking. While the saturation dynamics is hard to model at our experimental pressure condition (intermediate between homogeneous and inhomogeneous line broadening), it is easily characterized for a given experimental configuration. A linear correction is found to be sufficient and stable in time for measurements in the entire range of 0.2 ppt to 20 ppb useful for most applications. At larger concentration levels, a nonlinear correction could be easily adopted as well. Optical saturation can be lowered, either by increasing the gas pressure (at the cost of a loss in sensitivity) or by decreasing the laser intensity using cavity mirrors of larger focal length to increase the laser beam waist inside the resonant cavity (at the cost of an increase of the gas sample volume).

Today, a few instruments have been optimized to reach a sub-ppb NO detection limit, and three of them reach a LOD lower than 10 ppt ([16,18] and this work). These results should stimulate an in-depth investigation and an inter-comparison of instruments that were developed with different techniques to assess the accuracy and stability of NO measurements at very low concentrations.
